# High-Resolution Genomic Profiling of Carbapenem-Resistant *Klebsiella pneumoniae* Isolates: A Multicentric Retrospective Indian Study

**DOI:** 10.1093/cid/ciab767

**Published:** 2021-12-01

**Authors:** Geetha Nagaraj, Varun Shamanna, Vandana Govindan, Steffimole Rose, D Sravani, K P Akshata, M R Shincy, V T Venkatesha, Monica Abrudan, Silvia Argimón, Mihir Kekre, Anthony Underwood, David M Aanensen, K L Ravikumar, NIHR Global Health Research Unit on Genomic Surveillance of Antimicrobial Resistance, Khalil Abudahab, Harry Harste, Dawn Muddyman, Ben Taylor, Nicole Wheeler, Sophia David, Pilar Donado-Godoy, Johan Fabian Bernal, Alejandra Arevalo, Maria Fernanda Valencia, Erik C D Osma Castro, K N Ravishankar, Iruka N Okeke, Anderson O Oaikhena, Ayorinde O Afolayan, Jolaade J Ajiboye, Erkison Ewomazino Odih, Celia Carlos, Marietta L Lagrada, Polle Krystle V Macaranas, Agnettah M Olorosa, June M Gayeta, Elmer M Herrera

**Affiliations:** 1 Central Research Laboratory, Kempegowda Institute of Medical Sciences, Bengaluru, India; 2 Centre for Genomic Pathogen Surveillance, Big Data Institute, University of Oxford, Oxford, UK; 3 Wellcome Genome Campus, Hinxton, UK

**Keywords:** blaOXA232, carbapenem resistance, ColKP3, *K. pneumoniae*, KL51, ST231

## Abstract

**Background:**

Carbapenem-resistant *Klebsiella pneumoniae* (CRKP) is a threat to public health in India because of its high dissemination, mortality, and limited treatment options. Its genomic variability is reflected in the diversity of sequence types, virulence factors, and antimicrobial resistance (AMR) mechanisms. This study aims to characterize the clonal relationships and genetic mechanisms of resistance and virulence in CRKP isolates in India.

**Materials and Methods:**

We characterized 344 retrospective *K. pneumoniae* clinical isolates collected from 8 centers across India collected in 2013–2019. Susceptibility to antibiotics was tested with VITEK 2. Capsular types, multilocus sequence type, virulence genes, AMR determinants, plasmid replicon types, and a single-nucleotide polymorphism phylogeny were inferred from their whole genome sequences.

**Results:**

Phylogenetic analysis of the 325 *Klebsiella* isolates that passed quality control revealed 3 groups: *K. pneumoniae sensu stricto* (n = 307), *K. quasipneumoniae* (n = 17), and *K. variicola* (n = 1). Sequencing and capsular diversity analysis of the 307 *K. pneumoniae sensu stricto* isolates revealed 28 sequence types, 26 K-locus types, and 11 O-locus types, with ST231, KL51, and O1V2 being predominant. *bla*_OXA-48-like_ and *bla*_NDM-1/5_ were present in 73.2% and 24.4% of isolates, respectively. The major plasmid replicon types associated with carbapenase genes were IncF (51.0%) and Col group (35.0%).

**Conclusion:**

Our study documents for the first time the genetic diversity of K and O antigens circulating in India. The results demonstrate the practical applicability of genomic surveillance and its utility in tracking the population dynamics of CRKP. It alerts us to the urgency for longitudinal surveillance of these transmissible lineages.

*Klebsiella pneumoniae* is a common cause of nosocomial and community-acquired infections in newborns, the elderly, and immunocompromised patients [1]. The ability of *K. pneumoniae* to adapt by gene transfer, its virulence, and the convergence of resistance have led to the emergence of strains causing severe and untreatable invasive infections. Carbapenem-resistant *K. pneumoniae* (CRKP) is one of the most important and challenging pathogens causing infections with high mortality [2]. Cooccurrence of multidrug resistance (MDR) and hypervirulence among *K. pneumoniae* lineages has elicited concerns from a public health standpoint [3].

Epidemic lineages of CRKP have increasingly emerged and spread through global healthcare systems since they were first identified in 2001 [4]. The worldwide spread of these strains is alarming because they are MDR, with resistance to β-lactam antibiotics, fluoroquinolones, and aminoglycosides [5]. The resistance of these species is generally due to the production of carbapenemases, such as the class A serine carbapenemase *K. pneumoniae* (KPC) and metallo-β-lactamases. Other causes include combinations of outer membrane permeability loss and extended spectrum β-lactamase production [6].

According to the Center for Disease Dynamics, Economics and Policy, India has seen an increase in carbapenem resistance in *K. pneumoniae*, from 24% in 2008 to 59% in 2017, though several single-center studies have shown variable rates [7-9]. This dramatic increase in recent years is attributed, among other factors, to the lack of formal infection control measures and adequate medical intervention, prolonged hospitalization, the presence of comorbidities, and the overuse of antibiotics. In a single-center study from south India, the rate of mortality among patients with CRKP bloodstream infections is as high as 68% [10]. Despite the high disease burden, there are limited reports from India on the resistance mechanisms in MDR *K. pneumoniae* isolates, highlighting the need for comprehensive epidemiological surveillance results [10].

The expansion and dissemination of CRKP at different geographical locations throughout India necessitates a targeted analysis of the population structure, genomic mechanisms of resistance, and virulence of strains collected from the area of interest. Recent developments in understanding population structure highlight enormous genomic diversity and provide a basis for the pathogen to be tracked [11]. Whole genome sequencing (WGS) is a powerful tool for the characterization and surveillance of pathogens. It offers an unparalleled opportunity to explore genomic content and verify and evaluate the diversity of clusters and the frequency of contemporary isolates. It is now well-positioned to become the gold standard to resolve the knowledge gap [12].

Understanding of the mechanism by which this bacterium causes various infections is still basic, and most studies have limitations because of the narrow range of virulence factors being investigated. Little is known about the epidemiology of the capsule because serological and molecular typing are not commonly available, and several isolates are not typable using these methods. Phenotypic studies have identified 77 distinct capsule types (K types) and genomic studies have identified 134, but the true extent of capsule diversity remains unknown [13]. WGS can provide new insights into disease transmission, virulence, and antimicrobial resistance (AMR) dynamics when combined with epidemiological, clinical, and phenotypic microbiological information [12].

Since 2013, the Central Research Laboratory, Kempegowda Institute of Medical Sciences in India has developed a network of tertiary care hospitals, medical college hospitals, and stand-alone diagnostic laboratories across India. This network was extended for collection of retrospective isolates belonging to World Health Organization priority bacterial pathogens. In this report, we characterize the clonal relationships and genetic mechanisms of resistance and virulence in CRKP isolates in India. WGS was performed on a retrospective collection of 344 *K. pneumoniae* isolates to characterize their relationships, multilocus sequence type (MLST), capsular type, virulence genes, and AMR determinants.

## MATERIALS AND METHODS

### Bacterial Isolates and Phenotypic Characterization

The study included 344 retrospective (2013–2019) invasive and noninvasive putative *K. pneumoniae* isolates received from 8 teaching hospitals in 6 Indian regions. They were characterized at Central Research Laboratory, Kempegowda Institute of Medical Sciences, using the VITEK 2 (Biomeurieux) compact system. The results were interpreted according to the 2019 The Clinical and Laboratory standards Institute guidelines. Isolates with phenotypic carbapenem resistance of resistant (R) or intermediate (I) are considered resistant (R). An isolate is designated MDR when it shows resistance to ≥ 1 agent in ≥ 3 antimicrobial categories [12].

### Sequencing and Genomic Analyses

Genomic DNA was extracted from bacterial isolates with Qiagen QIAamp DNA Mini kit, in accordance with the manufacturer’s instructions. Double-stranded DNA libraries with 450 bp insert size were prepared and sequenced on the Illumina platform with 150 bp paired-end chemistry.

Bioinformatic analysis was performed using Nextflow pipelines developed as a part of Genomic Surveillance of Antimicrobial Resistance-AMR as detailed in www.protocols.io [14]. The genomes of 325 samples that passed sequence quality control were assembled using Spades v3.14 to generate contigs and annotated with Prokka v1.5 [15, 16]. The species identification was done using bactinspector and contamination was assessed using confindr [17, 18]. All the quality metrics were combined using multiqc and qualifyr to generate web-based reports [19, 20]. MLST and AMR and virulence factors were identified using ARIBA tool v2.14.4 [21] with BIGSdb-Pasteur MLST database [22], NCBI AMR acquired gene and PointFinder databases [20] and VFDB [23], respectively (Supplementary Table 1).

SNPs were identified for 307 *K. pneumoniae* isolates by mapping of reads to the NCBI reference genome, *K. pneumoniae* strain NTUH-K2044, NC_006625.1 using bwa mem [24], and the variants called were filtered and a maximum likelihood phylogeny was constructed with 1000 bootstrap support using IQTree [25] implemented in SNP phylogeny pipeline [26].

K and O antigen types, virulence factors, and plasmid replicons specific to *Klebsiella* species were identified using the kleborate and Kaptive implemented in the Pathogenwatch [27].

## RESULTS AND DISCUSSION

### Summary of the Collection

The collection consisted of 325 isolates from patients aged 7 days to 96 years, of whom 60% were 50–80 years old. Most isolates were from urine (31.4%) and blood (29.8%) (Supplementary Table 2).

VITEK 2 Compact was used to identify 325 isolates as *K. pneumoniae*, and these were reassigned to the species *K. pneumoniae* (n = 307, 94.4%), *K. quasipneumoniae* (n = 17, 5.5%), and *K. variicola* (n = 1, 0.1%) by sequencing. This highlights the limitation of traditional identification methods to distinguish species within the *K. pneumoniae* species complex. In addition, the use of genomic tools unmasks the true clinical significance of each phylogroup and their potential epidemiological specificities [28].

### Clonal Distribution

Clonal lineages of *K. pneumoniae* differ in their ability to acquire resistance and virulence genes, and in their propensity to spread within hospital and community environments [29]. Overall, the *K. pneumoniae* isolates sequenced in this study belonged to 28 different sequence types (STs), with ST231 being the most common sequence type (n = 107, 34.8%), followed by ST147 (n = 73, 23.5%) and ST14 (n = 26, 8.5%), accounting for 67.1% of total *K. pneumoniae* isolates (Table 1, Supplementary Figure 1). High prevalence of ST231 is in concordance with published data from India [30]. ST231 was the only clonal lineage represented by different centers and different specimen sources across India in the our and other Indian studies [10, 30]. Temporal distribution of ST231 isolates throughout the study period (2014–2019) from different regions of India demonstrates its regional distribution and spread across India. Although ST147 and ST14 were observed in 4 study centers (north, south, and western part of India). *K. pneumoniae* isolates from a hospital in the western region of India had 22 STs, revealing polyclonality within a single hospital.

**Table 1.** Distribution of AMR and Virulence Genes Across Top 5 STs Prevalent in K. pneumoniae Isolates From India

ST	No. of Isolates	KL Loci	O Loci	Virulence	Plasmids	Carbapenem Genes	ESBL Genes	Genomic AMR (%)
ST231	107	KL51 = 104 (98.1%), KL64 = 2 (1.9%)	O1v2 = 104 (98.1%), O1v1 = 1 (0.9%), O2v2 = 1 (0.9%)	Yersiniabactin = 106, aerobactin = 99, enterobactin = 106, type 1 fimbriae = 106, type 3 fimbriae = 106	IncFIB (pQil) = 106, IncFIB (K) = 3, Col440I = 104, ColKP3 = 97, IncFII (K) = 106, IncR = 2	blaOXA232 = 97, blaNDM1 = 1	blaSHV-12 = 3, blaCTX-M-15 = 95	Aminoglycoside = 100 (94%), beta-lactam = 107 (100%), carbapenem = 96 (91%), cephalosporin = 98 (92%), chloramphenicol = 107 (100%), fosfomycin = 107 (100%), phenicol/quinolone = 107 (100%), sulfonamide = 98 (92%), macrolide = 100 (94%), trimethoprim = 102 (96%), tetracycline = 2 (2%)
ST147	73	KL64 = 64 (87%), KL10 = 6 (8.6%), KL107 = 1 (1.4%), KL51 = 1 (1.4%), KL81 = 1 (1.4%)	O2v1 = 63 (86.3%), O3 = 6 (8.2%), O2v2 = 1 (1.4%), O101 = 2 (2.7%)	Yersiniabactin = 60, enterobactin = 28,type 1 fimbriae = 73, type 3 fimbriae = 73	IncFIB (pQil) = 6, IncFIB (K) = 2, Col440I = 67, ColKP3 = 45, IncFII (K) = 6, IncR = 67	blaNDM-1 = 5, blaOXA-181 = 43, blaNDM-5 = 9, blaOXA-232 = 7	blaSHV-12 = 1, blaCTX-M-15 = 66	Aminoglycoside = 60 (87%), beta-lactam = 73 (100%), carbapenem = 58 (84%), cephalosporin = 68 (99%), chloramphenicol = 63 (91%), fosfomycin = 73 (100%), phenicol/quinolone = 73 (100%), sulfonamide = 28 (41%), macrolide = 61 (88%), trimethoprim = 64 (93%), tetracycline = 4 (6%)
ST14	26	KL2 = 21 (80%), KL64 = 5 (20%)	O1v1 = 21 (80%), O1/O2v1 = 5 (20%)	Yersiniabactin = 26, aerobactin = 4, rmpa2 = 4, enterobactin = 26, type 1 fimbriae = 26, type 3 fimbriae = 26	IncFIB (pQil) = 1, IncFIB (K) = 22, Col440I = 5, ColKP3 = 15, IncFII (K) = 16, IncR = 15	blaNDM-5 = 6, blaOXA-232 = 14, blaNDM-1 = 6, blaOXA-181 = 3	blaCTX-M-15 = 26	Aminoglycoside = 8 (31%), beta-lactam = 26 (100%), carbapenem = 20 (77%), cephalosporin = 26 (100%), chloramphenicol = 20 (77%), fosfomycin = 26 (100%), phenicol/quinolone = 26 (100%), sulfonamide = 22 (85%), macrolide = 22 (85%), trimethoprim = 26 (100%), tetracycline = 11 (42%)
ST395	18	KL64 = 18 (100%)	O1v1 = 18 (100%)	Yersiniabactin = 18, rmpa2 = 1, enterobactin = 18, type 1 fimbriae = 18, type 3 fimbriae = 18	IncFIB (pQil) = 18, IncFIB (K) = 0, Col440I = 18, ColKP3 = 12	blaNDM-5 = 9, blaOXA-232 = 9, blaOXA-181 = 1	blaSHV-12 = 6, blaCTX-M-15 = 14	Aminoglycoside = 9 (50%), beta-lactam = 11 (61%), carbapenem = 12 (67%), cephalosporin = 11 (61%), chloramphenicol = 10 (56%), fosfomycin = 14 (78%), phenicol/quinolone = 14 (78%), sulfonamide = 10 (56%), macrolide = 10 (56%), trimethoprim = 12 (67%), tetracycline = 0(0%)
ST2096	12	KL64 = 12 (100%)	O1v1 = 12 (100%)	Yersiniabactin = 12, aerobactin = 10, rmpa2 = 10, enterobactin = 12, type 1 fimbriae = 12, type 3 fimbriae = 12	IncFIB (K) = 12, Col440I = 12, ColKP3 = 10, IncFII (K) = 9, IncR = 1	blaOXA-232 = 11	blaCTX-M-15 = 10	Aminoglycoside = 1 (8%), beta-lactam = 10 (83%), carbapenem = 11 (91.6%), cephalosporin = 10 (83%), chloramphenicol = 7 (58%), fosfomycin = 10 (83%), phenicol/quinolone = 10 (83%), sulfonamide = 8 (67%), macrolide = 8 (67%), trimethoprim = 10 (83%), tetracycline = 8 (67%)
ST16	10	KL51 = 5(50%), KL81 = 4 (40%), KL48 = 1 (10%)	O3b = 5 (50%), O101 = 4 (40%), O2v1 = 1(10%)	Yersiniabactin = 9, aerobactin = 2, rmpa2 = 1, enterobactin = 10, type 1 fimbriae = 10, type 3 fimbriae = 10	IncFIB(K) = 9, Col440I = 5, ColKP3 = 3, IncFII (K) = 10, IncR = 4	blaNDM-5 = 4, blaOXA-181 = 4, blaOXA-232 = 3, blaNDM-1 = 1	blaCTX-M-15 = 10	Aminoglycoside = 8 (80%), beta-lactam = 6 (60%), carbapenem = 8 (80%), cephalosporin = 9 (90%), chloramphenicol = 4 (40%), fosfomycin = 9 (90%), phenicol/quinolone = 9 (90%), sulfonamide = 9 (90%), macrolide = 8 (80%), trimethoprim = 9 (90%), tetracycline = 4 (40%)
ST437	10	KL36 = 10 (100%)	O4 = 10 (100%)	Yersiniabactin = 10, enterobactin = 10, type 1 fimbriae = 10, type 3 fimbriae = 10	IncFIB(pQil) = 10, IncFIB(K) = 0Col440I = 10, ColKP3 = 10	blaNDM-5 = 10, blaOXA-232 = 10	blaCTX-M-15 = 10	Aminoglycoside = 10 (100%), beta-lactam = 10 (100%), carbapenem = 10 (100%), cephalosporin = 10 (100%), chloramphenicol = 0 (0%), fosfomycin = 10 (100%), phenicol/quinolone = 10 (100%), sulfonamide = 10 (100%), macrolide = 10 (100%), trimethoprim = 10 (100%), tetracycline = 0(0%)
ST469	9	KL139 = 9 (100%)	O3b = 9 (100%)	Enterobactin = 9, type 1 fimbriae = 9, type 3 fimbriae = 9	IncFIB(K) = 9	…	…	Aminoglycoside = 0 (0%), beta-lactam = 0 (0%), carbapenem = 0 (0%), cephalosporin = 9 (100%), chloramphenicol = 0 (0%), fosfomycin = 9 (100%), phenicol/quinolone = 9 (100%), sulfonamide = 0 (0%), macrolide = 0 (0%), trimethoprim = 0 (0%), tetracycline = 0 (0%)
ST38	6	KL52 = 6 (100%)	O101 = 6 (100%)	Yersiniabactin = 6, enterobactin-6, type 1 fimbriae = 6, type 3 fimbriae = 6	IncFIB (pQil) = 5 IncFIB (K) = 6, Col440I = 6, ColKP3 = 6, IncFII (K) = 6	blaNDM-5 = 1, blaOXA-232 = 6, blaNDM-1 = 5	blaCTX-M-15 = 6	Aminoglycoside = 6 (100%), beta-lactam = 6 (100%), carbapenem = 6 (100%), cephalosporin = 6 (100%),chloramphenicol = 6 (100%), fosfomycin = 6 (100%), phenicol/quinolone = 0 (0%), sulfonamide = 6 (100%), macrolide = 0 (0%), trimethoprim = 6 (100%), tetracycline = 6 (100%)
ST11	4	KL81 = 1 (25%), KL2 = 1 (25%), KL24 = 1 (25%), KL15 = 1 (25%)	O2v1 = 2 (50%), O101 = 1 (25%), O4 = 1 (25%)	Yersiniabactin = 4, enterobactin = 4, type 1 fimbriae = 4, type 3 fimbriae = 4	IncFIB (pQil) = 2, IncFIB (K) = 2, Col440I = 1, ColKP3 = 1, IncFII (K) = 3, IncR = 3	blaNDM-5 = 1, blaOXA-232 = 1, blaNDM-1 = 1	blaCTX-M-15 = 3	Aminoglycoside = 2 (50%), beta-lactam = 3 (75%), carbapenem = 2 (50%), cephalosporin = 3 (75%), chloramphenicol = 3 (75%), fosfomycin = 3 (75%), phenicol/quinolone = 2 (50%), sulfonamide = 2 (50%), macrolide = 2 (50%), trimethoprim = 2 (50%), tetracycline = 0 (0%)

Abbreviations: AMR, antimicrobial resistance; ESBL, extended spectrum beta lactamase; ST, sequence type.

ST258 is recognized as the most prevalent and extensively disseminated KPC-producing *K. pneumoniae* in many countries, which made its absence in our collection noteworthy. ST11, a single-locus variant of ST258 and a prevalent clone associated with the spread of KPC in Asia (particularly in China and Taiwan), was identified in 1.3% of the *K. pneumoniae* isolates [31]. ST147 and ST14, described as international high-risk clones associated with extensive drug resistance, accounted for 23.5% and 8.5% of the isolates in this study, respectively [32, 33]. One novel ST (ST5603) with extensive drug resistance was identified in a single *K. pneumoniae* isolate (Supplementary Table 2). The novel ST (gapA2-infB1-Mdh1-Pgi8-phoE10-rpoB4-tonb202) was a single-locus variant of ST890, varying at the tonb gene (tonb61), and possessed the KL107 and O1v1 loci.

KL51 (n = 111/307, 36.1%) and KL64 (n = 104/307, 33.8%) were dominant K-loci types in this study, whereas KL1, KL2, KL5, KL20, KL17, KL51, KL54, KL57 and KL64 are the reported K-loci types from other Indian studies [31]. KL51 is reported from isolates from the United States, Sweden, United Kingdom [34], Thailand [35], and Lebanon [36]. The phylogenetic tree of the 307 isolates shows the correlation between capsular locus type and sequence type, for example of KL51 with ST231, and of KL64 with ST147 and ST395 (Supplementary Figure 1). Notably, no isolates were assigned to KL1, despite its high prevalence in a previous study [37].

Of 11 O serotypes identified, O1, O2, and O3 together accounted for 90.2% of 307 *K. pneumoniae* samples (Supplementary Table 1). Strikingly, the O3b locus, which is considered rare, was detected in 5.2% (16/307) of the isolates [37]. Other serotypes present included O4 (4.5%), O locus 101 (4.5%), O locus 103 (0.33%), and O5 (0.33%). O locus types also showed ST-specific distribution. In particular, O1v1is found in STs 101, 1322, 14, 15, 2096, 231, 2497, 35, 48, and novel ST, whereas O2v2 is found in STs 307, 1248, 147, and 231. The stratification of O types by patient age established that O1, O2, and O3 account for 81% of samples in the age group ≤ 5 years (Supplementary Table 4).

### Virulence

More than 10 virulence factors account for the pathogenesis of CRKP, and their detection helps understand the pathogenesis of different strains [38]. In the present study, a total of 33 genes belonging to 6 major virulence factors were observed. The core virulence genes type I and III fimbriae, enterobactin, AcrAB efflux pump, and regulators (RmpA, RcsAB) were identified in all 307 isolates. The acquired virulence genes coding for colibactin, nutrition factor, salmochelin, and rmpA were completely absent. Yersiniabactin, an iron uptake locus (ybtAEPQSTUX), was identified in 89.9% (276/307) of the isolates, and the regulatory gene rmpA2 was present in 5.5% (17/307). Aerobactin, a dominant siderophore, was found in 38.1% (n = 117) of the isolates, all belonging to ST231 or ST2096 (Supplementary Figure 3). The highest recorded virulence score of 4 was observed in 117 isolates (38.1%), which carried yersiniabactin and aerobactin genes. Of the *K. pneumoniae* ST231-KL51 isolates, 94% were characterized by a virulence score of 4 (Supplementary Table 1). Phylogenetic analysis, using the dataset from this study and a global collection of ST231 isolates, identified a sublineage that has acquired aerobactin and yersiniabactin, as well as the OXA-232 carbapenemase [39].

### Resistance Profile and Their Distribution

Accumulation of AMR in *K. pneumoniae* is primarily the result of horizontal gene transfer aided by plasmids and mobile genetic elements. Since the first report of CRKP in 1996, the incidence of this MDR pathogen has increased significantly. The resistance is primarily due to production of acquired carbapenemases *bla*_KPC_, *bla*_OXA_, *bla*_NDM_, and the combinatorial mechanism of extended spectrum beta lactamase (ESBL) activity with loss of outer membrane porins [40]. This has become worrisome, particularly at a time when no new promising antimicrobial agents are on the horizon [41]. For public health initiatives, understanding their emergence and distribution over a diverse geographical region is needed [11].

**Figure 1.**
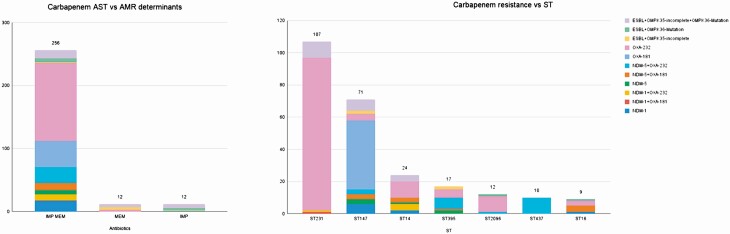
Carbapenem resistance mechanisms by ST. (A) Mechanisms of resistance to carbapenems identified in the genomes of 280 isolates grouped by their phenotypic carbapenem resistance profile. Genes responsible for carbapenems are indicated. (B) The determinants present in each ST and responsible for carbapenem resistance. Note, where more than 1 determinant is present, the single determinant or combination likely to result in the highest increased in susceptibility to carbapenems is recorded. ST, sequence type.

A total of 307 *K. pneumoniae* isolates displayed phenotypic resistance to ampicillin (99%), gentamicin (78.8%), amikacin (73%), amoxicillin-clavulanate (91.9%), piperacillin-tazobactam (93.5%), cefepime (92.2%), ceftriaxone (91.9%), ciprofloxacin (95.4%), carbapenems (91.2%), cotrimoxazole (87.9%), and colistin (8.8%). We observed 85 different resistance profiles in *K. pneumoniae* isolates (Supplementary Table 3). Of the 307 isolates, 280 were carbapenem-resistant. The majority of these showed phenotypic resistance to both meropenem and imipenem (256/280, 91.4%, Figure 1A), explained by the presence of *bla*_OXA-48-like_ genes, *bla*_NDM_ genes, and a combination of ESBLs (*bla*_CTX-M-15_, *bla*_CTX-M-71_, *bla*_SHV-12_, *bla*_SHV-13_, *bla*_SHV-27_, *bla*_SHV-31_, *bla*_SHV-41_) and inactivation of porins Ompk35/36 [45]. Of the carbapenem-resistant isolates, 80.3% (225/280) carried *bla*_OXA-48-like_ genes (*bla*_OXA181_ and *bla*_OXA232_), and 26.7% (75/280) carried *bla*_NDM-1/5_, which supports the reported changing trends in carbapenem resistance [42]. Of the 225 isolates with *bla*_OXA48-like_ genes, 60.7% (170/280) carried *bla*_OXA232_, and 19.6% (55/280) carried *bla*_OXA181_. *bla*_OXA232_ was present mostly within ST231 (97/170), in line with a previous report of widely dissemination of carbapenem-resistant ST231 with *bla*_OXA232_ across India (Figure 1B) [30]. Notably, no *bla*_KPC_ genes were detected, which are the predominant carbapenemases found in Europe and in Colombia [29, 43].

In the present dataset, resistance to carbapenems is also conferred by ESBLs when combined with porin loss-of-function mutations [44]. Disruption of the Ompk35 porin (n = 231) was observed in the study isolates, and insertion mutations of TD/GD amino acids at position 115 of Ompk36 gene were found (n = 224). The disruption of major outer membrane protein genes (Ompk35/Ompk36) along with ESBLs (*bla*_CTX-M-15_, *bla*_CTX-M-71_, *bla*_SHV-12_, *bla*_SHV-13_, *bla*_SHV-27_, *bla*_SHV-31_, *bla*_SHV-41_) were detected in 92.1% of the isolates (258/280). The presence of multiple ESBL genes in a single isolate (n = 12), highlights the emerging complexity of antibacterial resistance repertoire.

### Plasmid Repertoire

The predominant plasmid replicons present in the 307 *K. pneumoniae* isolates are Col440I (79.2%), ColKP3 (68.7%), and IncFII (K) (57.0%) (Supplementary Figure 4). Other plasmid replicons detected are IncX, IncH, IncL, IncA/C, IncY, and IncQ. Understanding such vectors carrying the AMR genes could help in improving strategies to control AMR dissemination.

In the collection, 225 isolates carried *bla*_OXA48-like_ genes. We observed that 93.7% (n = 211) of them harbored a plasmid with the ColKP3 replicon sequence (Supplementary Figure 2). A similar association between the ColKP3 replicon and the *bla*_OXA-232_ gene was also observed in ST231 isolates from around the world (Supplementary Figure 5) [39].

### High-Risk Clone: ST231

ST231, an emerging CRKP epidemic clone, was reported from India, France, Singapore, Brunei, Darussalam, and Switzerland [45]. In southeast Asia, this clone was found to be MDR, combining resistance to carbapenems, extended-spectrum cephalosporins, and broad-spectrum aminoglycosides [46]. In India, ST231 was first reported in 2013 in Delhi, with the isolate carrying *bla*_OXA-232_ as the predominant *bla*_OXA48-like_ carbapenemase variant [30].

**Figure 2.**
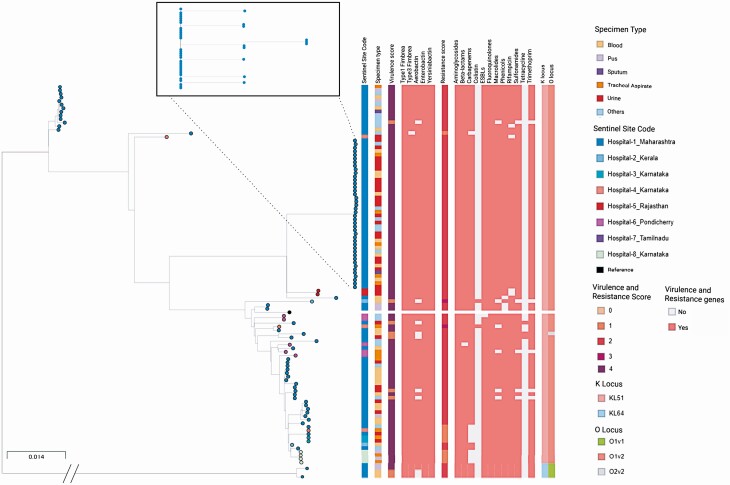
Phylogenetic analysis of ST231 lineage. A phylogenetic tree of 107 ST231 genomes from India. The lineage highlighted in the box is shown in detail, with metadata blocks showing ST, K locus, AMR determinants, and virulence factors. The full data are available at https://microreact.org/project/5GDXCkZsvajcXTmW89LRED/746bb511. AMR, antimicrobial resistance; ST, sequence type.

Isolates belonging to ST231 (n = 107) were entirely genotypically MDR. Of 107 ST231 isolates, 89 were from hospital 1 (western part of India), 16 isolates were from 5 centers from the southern part of India, and 2 isolates were from the northern part of India. Of the ST231 isolates, 91.5% (n = 98/107) carried *bla*_OXA-232_ genes and 99.0% (n = 106/107) carried porin (Ompk35/Ompk36) mutations and ESBL genes (*bla*_CTX-M-15_, *bla*_SHV-12_), a possible mechanism for nonsusceptibility to carbapenems (Figure 2). In addition, ST231 genomes harbored other resistance genes, including rmtF (39/107, aminoglycoside resistance), ermB (98/107, macrolide resistance), catA1 (104/107, phenicol resistance), sul1 (101/107, sulfonamide resistance), dfrA12 (101/107, trimethoprim resistance), and mutations in gyrA_83I and parC_80I (107/107, fluoroquinolone resistance).

When compared with global genomes, the ST231 genomes from our study and those from a previous study in India (BioProject accession number PRJEB30913) shared a common repertoire of AMR and virulence determinants, and were found interspersed with (and often basal to) genomes from other countries in the tree, thus pointing to international dissemination (Supplementary Figure 5) [39, 47].

The tree of 107 ST231 isolates from this study showed evidence of clonal spread of ST231 carrying *bla*_OXA-232_ and *bla*_CTX-M-15_ within 1 hospital over a period of 3 years (hospital 1, Figure 2). Forty-two isolates formed a tight cluster with a mean SNP difference of 1 (range, 0–3), and they were separated by at least 43 SNP differences from the remaining 65 ST231 isolates in this study. The isolates in this cluster were mostly from inpatients (37/42) and characterized by a median patient age of 73.5 years (range, 26–89) compared with 67 years (range, 7 days–96 years) for all 239 *K. pneumoniae* isolates collected by this hospital. Altogether, this reveals a persistent outbreak of carbapenem- and cephalosporin-resistant ST231 within hospital 1 and underscores the need to strengthen infection prevention and control. Importantly, the tree also shows other ST231 genomes from hospital 1 that show similar resistance and virulence profiles, but that can be clearly distinguished from this large outbreak by their clustering, highlighting the utility of WGS to rule cases out of an outbreak investigation even when other phenotypic or genotypic markers would be inconclusive.

We conclude that the MDR ST231 lineage carrying both important resistance and virulence determinants is a major and rapidly disseminating CRKP high-risk clone in India capable of causing nosocomial outbreaks [30]. The emergence of the ST231 clonal lineage has also recently been reported in Switzerland, France, and Thailand [48-50]. The presence of MDR and virulence genes poses a risk in that the lineage may be a reservoir of virulence-associated genes that can be passed on by horizontal gene transfer to other lineages. This means that a high level of vigilance and monitoring is required. As shown by other studies, WGS can be used as an outbreak detection tool, allowing the detection of widely dispersed outbreaks that might not be otherwise identified.

This study has some limitations. First, very few retrospective isolates were retrieved from 7 sentinel sites and majority of the isolates were from 1 hospital, which was an archival facility.

Furthermore, the outbreak observed here were sampled from 1 center. However, this study represents a starting point for deeper understanding of *K. pneumoniae* population structure and diversity and we will build on these findings with prospective genomic surveillance connecting more hospitals representing each of the Indian states.

## CONCLUSION

The study establishes the presence of several high-risk MDR CRKP clones in clinical samples collected across India. It represents the basis for genomic surveillance of emerging CRKP in India, providing critical information that can be used to track the emergence and dissemination, and assess the potential impact, of important variants. The lack of structured surveillance framework and inability to access patient clinical data has limited our interpretation. To the best of our knowledge, this is the first WGS study from India to document genetic diversity of K and O antigens circulating in Indian CRKP isolates.

## Supplementary Data

Supplementary materials are available at *Clinical Infectious Diseases* online. Consisting of data provided by the authors to benefit the reader, the posted materials are not copyedited and are the sole responsibility of the authors, so questions or comments should be addressed to the corresponding author.

ciab767_suppl_Supplementary_Material

ciab767_suppl_Supplementary_Table_1

## Notes

***Financial support.*** This work was supported by Official Development Assistance (ODA) funding from the National Institute for Health Research [grant number 16_136_111] and the Wellcome Trust grant number 206194.

This research was commissioned by the National Institute for Health Research using Official Development Assistance (ODA) funding. The views expressed in this publication are those of the authors and not necessarily those of the NHS, the National Institute for Health Research or the Department of Health.

***Conflict of interest.*** The authors: No reported conflicts of interest. All authors have submitted the ICMJE Form for Disclosure of Potential Conflicts of Interest. M. K. reports support from Centre for Genomic Pathogen Surveillance (CGPS), during the conduct of the study; reports grants, travel support, and receipt of equipment, materials, drugs, medical writing, gifts, or other services from National Institute for Health Research (NIHR). V.G., V.T.V., G.N., D.S., V.S., M.R.S., S.R., K.P.A., and K.L.R. report travel support from NIHR, UK. M.A. reports support from Wellcome Connecting Science during the conduct of the study.

***Supplement sponsorship.*** The supplement is sponsored by the UK National Institute for Health Research Global Health Research Unit on Genomic Surveillance of AMR.

***Acknowledgments.*** Members of the NIHR Global Health Research Unit on Genomic Surveillance of Antimicrobial Resistance: Khalil Abudahab, Harry Harste, Dawn Muddyman, Ben Taylor, Nicole Wheeler, and Sophia David of the Centre for Genomic Pathogen Surveillance, Big Data Institute, University of Oxford, Old Road Campus, Oxford, UK, and Wellcome Genome Campus, Hinxton, UK; Pilar Donado-Godoy, Johan Fabian Bernal, Alejandra Arevalo, Maria Fernanda Valencia, and Erik C. D. Osma Castro of the Colombian Integrated Program for Antimicrobial Resistance Surveillance, Coipars, CI Tibaitatá, Corporación Colombiana de Investigación Agropecuaria (AGROSAVIA), Tibaitatá, Mosquera, Cundinamarca, Colombia; K. N. Ravishankar of the Central Research Laboratory, Kempegowda Institute of Medical Sciences, Bengaluru, India; Iruka N. Okeke, Anderson O. Oaikhena, Ayorinde O. Afolayan, Jolaade J Ajiboye, and Erkison Ewomazino Odih of the Department of Pharmaceutical Microbiology, Faculty of Pharmacy, University of Ibadan, Oyo State, Nigeria; Celia Carlos, Marietta L. Lagrada, Polle Krystle V. Macaranas, Agnettah M. Olorosa, June M. Gayeta, and Elmer M. Herrera of the Antimicrobial Resistance Surveillance Reference Laboratory, Research Institute for Tropical Medicine, Muntinlupa, the Philippines; Ali Molloy, alimolloy.com; John Stelling, The Brigham and Women’s Hospital, Boston, MA, USA; and Carolin Vegvari, Imperial College London, London, UK.
